# Project BETTER: A Family-Centered, Technology-Delivered Intervention for Pregnant People with Opioid Use Disorder

**DOI:** 10.3390/children10020359

**Published:** 2023-02-11

**Authors:** Anna Beth Parlier-Ahmad, Michelle Eglovitch, Sarah Martin, Dace S. Svikis, Caitlin E. Martin

**Affiliations:** 1 Department of Psychology, Virginia Commonwealth University, Richmond, VA 23284, USA; 2 School of Medicine, Virginia Commonwealth University, Richmond, VA 23298, USA; 3 Institute for Drug and Alcohol Studies, Virginia Commonwealth University, Richmond, VA 23298, USA; 4 Department of Obstetrics and Gynecology, School of Medicine, Virginia Commonwealth University, Richmond, VA 23298, USA

**Keywords:** family-centered care, technology-delivered intervention, opioid use disorder, medication for opioid use disorder, maternal–infant dyad, neonatal opioid withdrawal syndrome, child welfare

## Abstract

Birthing people with opioid use disorder (OUD) face unique stressors during the transition from pregnancy to postpartum that can negatively impact the maternal–infant dyad. This study aimed to describe the development of a family-centered, technology-delivered intervention tailored to help pregnant people receiving medication for OUD (MOUD) prepare for this transition. Formative data from patients and providers identified intervention content: (1) recovery-oriented strategies for the pregnancy-to-postpartum transition; (2) guidance around caring for an infant with opioid withdrawal symptoms; and (3) preparation for child welfare interactions. The content was reviewed in successive rounds by an expert panel and modified. Pregnant and postpartum people receiving MOUD pre-tested the intervention modules and provided feedback in semi-structured interviews. The multidisciplinary expert panel members (*n* = 15) identified strengths and areas for improvement. Primary areas for improvement included adding content, providing more structure to help participants navigate the intervention more easily, and revising language. Pre-testing participants (*n* = 9) highlighted four themes: reactions to intervention content, navigability of the intervention, feasibility of the intervention, and recommendation of the intervention. All iterative feedback was incorporated into the final intervention modules for the prospective randomized clinical trial. Family-centered interventions tailored for pregnant people receiving MOUD should be informed by patient-reported needs and multidisciplinary perspectives.

## 1. Introduction

The prevalence of pregnancies affected by opioid use disorder (OUD) is rising [1]. Opioid overdose is a leading cause of pregnancy-associated deaths, largely due to unique challenges in the postpartum period [2,3,4]. Recent research among pregnant and parenting people with OUD identified stress, lack of social support, infant care, fear surrounding child welfare interactions, and mental health issues as common contributors to negative maternal–infant dyad outcomes including overdose [5].

Evidence-based treatment for OUD, including medication for OUD (MOUD), reduces risk for opioid use recurrence and overdose [6,7]. However, a significant gap exists between treatment need and availability [8], and MOUD discontinuation is common after infant delivery for individuals who do access treatment [9,10]. Common challenges to postpartum MOUD continuity are comorbid mental health conditions [9], stigma [11], and child welfare concerns [12]. Notably, postpartum and parenting people with OUD report being underprepared for the pregnancy-to-postpartum transition and care of infants who develop neonatal opioid withdrawal syndrome (NOWS); they desire family-centered resources to help address these gaps [13,14].

Novel tools to equip the maternal–infant dyad impacted by OUD for continued recovery and positive long-term outcomes are needed [15]. Evidence-based, technology-delivered interventions that focus on family-centered maternity care can supplement standard care to extend the reach of perinatal OUD interventions and relieve clinician burden [16,17,18]. Technology-based interventions have demonstrated effectiveness for parental education [19,20] and behavior change in vulnerable populations [21,22]. Evidence further supports the acceptability of technology-based screening and brief motivational interventions for substance use among pregnant and postpartum people [23,24].

Existing technology-delivered interventions for people with substance use disorder (SUD) adapted from gold standard therapist-delivered care models (e.g., cognitive behavioral therapy) are effective [17,25,26] but are not designed to address the unique needs of pregnant and parenting people with OUD during the postpartum period. To our knowledge, no technology-based interventions specific to the pregnancy-to-postpartum transition for people receiving MOUD have been developed and evaluated. The purpose of this paper is to describe the iterative development process of Project BETTER’s (Bringing Education Through Technology, Empathic listening, and Research) novel family-centered, technology-delivered educational intervention as it underwent refinement in preparation for evaluation in a prospective randomized clinical trial.

## 2. Methods

### 2.1. Initial Development of Project BETTER’s Intervention

Providers in obstetrics, pediatrics, and addiction medicine identified the clinical need to provide more robust preparation for the pregnancy-to-postpartum transition for birthing people receiving MOUD. Patient and provider qualitative input suggested three areas of need: (1) recovery-oriented care strategies for birthing people with OUD during the pregnancy-to-postpartum transition, (2) guidance around caring for an infant at risk for developing NOWS, and (3) preparation to interact with child welfare after delivery [27]. We created a study team consisting of clinical researchers across the specialties of obstetrics, addiction medicine, and psychology to develop an intervention to address these areas.

Using the formative data and aspects of evidence-based approaches, the study team drafted intervention content for three modules corresponding to the clinical need areas. The modules are based on principles of motivational interviewing (e.g., empathetic, non-confrontational, and person-centric) and include techniques such as open-ended questions, affirmations, reflective listening, and summaries [28]. Each module is guided by an interactive narrator (a synthetic text to speech engine) who reads questions and speaks tailored content aloud to participants. Psychoeducation is delivered via professionally produced videos featuring providers from across the spectrum of care and patient testimonials. Each module was designed to be completed once in approximately 20 min. The modules can be accessed indefinitely after completion.

### 2.2. Expert Panel Review and Project BETTER’s Intervention Refinement

The expert panel representing patient, provider, and researcher perspectives (*n* = 15) reviewed and critiqued the drafted intervention content in successive rounds. First, a group of 12 multidisciplinary providers from obstetrics, addiction medicine, pediatrics, nursing, psychology, social work, and child welfare, representing all components of patient care within the study clinic, reviewed the content. A patient then reviewed the content. Finally, a three-person team of experts in technology interventions for SUD reviewed the content. Overall, each area of expertise was represented by one to three experts. The reviewers were blinded to feedback from other reviewers. Feedback from the expert panel was compiled. The study team revised all materials incorporating expert panel feedback.

The intervention content for each module was then programmed into the Computerized Intervention Authoring System (CIAS), version 3.0 (Michigan State University, Michigan, United States, EB028990 (PI Ondersma))[29]. CIAS 3.0 is an open-source authoring tool that allows for the creation of electronic interventions without coding. Interventions built using CIAS are accessed through a unique link to the intervention website. CIAS 3.0 utilizes synchronous interactivity, reflections, branching logic, a clean user interface, and the ability to incorporate specific images, text, and videos. CIAS applications are HIPAA compliant and are hosted on a HIPAA-compliant cloud server.

### 2.3. Project BETTER’s Intervention Pre-Testing by Patients

Birthing people receiving MOUD at a perinatal addiction clinic pre-tested the technology-delivered intervention (*n* = 9; 3 per module) in August through October 2021. Patients enrolled in the clinic research registry who were at least 18 years of age, had OUD, were receiving MOUD, and were pregnant or had given birth within the past 12 months were invited to pre-test the intervention. Participants completed a brief demographic survey and were given the option to complete one of three intervention modules either in the clinical space using a provided tablet or remotely on their own electronic device. Upon completion of the module, participants completed a 15 minute semi-structured research assistant (RA)-led interview to provide feedback on the content and delivery of the module. The interviews assessed participants’ overall impressions, areas for improvement, opinions about learning in the technology-delivered format, feasibility of completing the modules during pregnancy, and likelihood of recommending to pregnant people receiving MOUD. The interviews were recorded and summarized to inform intervention revision. All participants provided verbal informed consent. The Institutional Review Board approved these procedures.

## 3. Results

### 3.1. Expert Panel Review and Project BETTER’s Intervention Refinement

The expert panel feedback was summarized by identified strengths and areas for improvement (Table 1). Panel members liked the innovative and engaging format of the intervention and the informative content. Primary areas for improvement included adding content (e.g., concrete problem solving and coping strategies), providing more structure to help participants navigate the intervention more easily (e.g., provide length of modules), and revising some language to be more accurate and inclusive. The study team addressed all suggested improvements in intervention revisions prior to patient pre-testing.

### 3.2. Project BETTER’s Intervention Pre-Testing by Patients

The participants (*n* = 9) were of reproductive age (30.4 ± 4.6 years), White (56%), American Indian (22%), and Black (11%). About two thirds were postpartum (67%), and all had a psychiatric comorbidity. Most were receiving buprenorphine for more than two years (78%). Feedback had four overarching themes: reactions to intervention content, intervention navigability, feasibility, and recommendation of the intervention (Table 2).

The participants voiced positive feelings toward the animated narrator and described feeling comfortable learning in this format, expressing that they could learn without feeling judged for their addiction. The participants noted that intervention content was helpful as a supplement to standard perinatal education and as a guide for discussions with providers. They found the modules to be navigable, with only a few participants experiencing technological challenges. The participants believed it would be feasible to complete the intervention during pregnancy and reported that they would recommend it to other pregnant people receiving MOUD.

The participants suggested shortening some narrator reflections and adding the RA contact information into the modules to improve the intervention; content was revised accordingly. One participant reported that she just “clicked through” the modules and was not attentive to the content. Therefore, attention check questions were added to each module.

## 4. Discussion

Despite high rates of OUD-related postpartum morbidity and mortality [3], evidence-based strategies promoting family-centered, recovery-oriented care are limited for birthing people receiving MOUD [14,27]. Tailored technology-delivered interventions may help address this gap. We describe the development of Project BETTER’s technology-delivered intervention in preparation for its evaluation in a prospective randomized clinical trial.

Evidence for the use of technology-based behavioral interventions for SUD assessment and treatment is substantial [16,17,30], and the provision of virtual prenatal care has expanded with the COVID-19 pandemic [31]. The findings from our patient pre-testing suggest that pregnant and postpartum people receiving MOUD are comfortable with and interested in Project BETTER’s family-centered, technology-delivered intervention. The positive feedback received is consistent with the literature underscoring the importance of engaging end-users (providers and patients) in the intervention development process [17].

Addressing stigma is essential in interventions designed for people with OUD, as it is associated with compromised treatment outcomes [32,33]. Pregnant and parenting people are particularly susceptible to OUD- and MOUD-related stigma [34,35]. Technology-delivered interventions may reduce stigma by using techniques such as narrator empathy [36]. Prior work has shown that postpartum people with SUD prefer using technology over talking with medical staff about their substance use [37]. Additionally, technology-based methods can improve reporting of socially undesirable behaviors and sensitive topics [38]. Like previous research, our participants reported reduced anxiety when discussing their addiction with the technology interface compared to in person.

Project BETTER’s intervention was based on patient-reported needs and multidisciplinary perspectives from one study site, limiting generalizability. However, Project BETTER’s intervention can be easily tailored to different geographic locations (e.g., rural areas) [39]. Future studies may include more expert panel members and/or additional expert groups such as a family support group. Additionally, the CIAS 3.0 software allows for continual intervention updates as the evidence base evolves. Pre-testing suggests that further assessment of Project BETTER’s intervention feasibility, acceptability, and effectiveness with a large sample in a clinical research setting is warranted.

## 5. Conclusions

Pregnant and postpartum people receiving MOUD are comfortable with educational technology-delivered interventions, such as Project BETTER’s intervention, that use formative data in the development process. The present description of development for a family-centered, technology-delivered intervention can aid in the development of similar interventions.

## Figures and Tables

**Table 1:** Expert panel feedback for Project BETTER’s novel technology-delivered intervention (*N* = 15).

Area of Expertise	Strengths	Areas for Improvement
Nursing	- Information is relevant to the audience and will promote engagement Animated narrator is appealing Interactive format	- Provide estimated module length Add definitions for terms Highlight prevalence of postpartum anxiety and mood disorders Encourage development of a postpartum self-care plan
Pediatrics	- Comprehensive content including medical and psychosocial aspects ‘Exciting’ format Modules can be completed across multiple ‘sessions’	- Encourage pediatric appointment attendance and proactive communication with healthcare team Add content about ways to cope with infant withdrawal symptoms Specify provider roles Add decreased sleep as a stressor
Peer recovery	- Provides opportunities to engage with provider through the intervention	- Review confidentiality of responses and provide a ‘prefer not to answer’ option for all questions Emphasize medical records autonomy
Social work	- Animated narrator is engaging	- Clarify child welfare terminology and process Clarify postpartum stress language
Clinical Psychology	- Provides considerable emphasis on self-care	- Use non-stigmatizing language (“support” instead of “help”) Acknowledge medical system distrust Consider using gender neutral language
Obstetrics and Addiction Medicine	- Topic check-ins are informative and engaging	- Acknowledge that strategies are helpful for some but not all people with opioid use disorder Add parents’ rights during child welfare interactions
Intervention Development	- Animated narrator has personality and confidence at some points in the intervention	- Increase animated narrator’s personality and confidence throughout Increase use of motivational interviewing techniques Introduce schema and add action plan Add description of intervention purpose to promote engagement Add “comments” box at the end
Child Welfare	- Offers substantial emphasis on promoting child wellbeing	- Include correct terminology regarding family assessments and investigations Provide strategies for actionable steps
Patient	- Range of different providers speaking in videos Personalized and interactive Relevant and helpful content	- Include a compassionate and nonjudgmental provider to discuss medication for opioid use disorder stigma Emphasize self-care and coping skills

**Table 2:** Qualitative findings from patient pre-testing of Project BETTER’s novel technology-delivered intervention (*N* = 9).

Themes	Sub-Themes	Example Quotations
Reactions to intervention content	Animated narrator well received	“I loved the parrot. The information from the little bird was adorable.”
	Reductions in negative feelings related to addiction stigma	“I wasn’t being judged and didn’t have to act a certain way and I could be myself while learning. I liked how y’all approached that, doing it with the bird instead of a person.” “I think as a mom when you’re already taking medication you already feel stigmatized judged and horrible for putting yourself and your baby through that. Reading about it or hearing it is better than having to talk to someone else.” “Sometimes when you suffer through addiction, you still feel like your doctor might be judging you a little bit even if they don’t say that or they don’t treat you that way, it’s a little less embarrassing without someone standing over you.”
	Learned new content through intervention	“A decent chunk of it was stuff I didn’t know. Like the question, how long are women on Suboxone after having the baby? I liked that part.”
	Helpful to guide discussions with providers	“Some people don’t know the questions to ask the providers. The modules hit on the topics then you can ask your providers. It would be a good idea to do that.” “While you’re in [the clinic] environment, it would be a great time to do it. It would give ideas about things you want to talk about or questions to bring up.”
Navigability of intervention	Easy to complete	“No, everything was easy- they give you the little button to press when you’re done with what you need to do. It was simple. I’m not good with technology and it was really easy.”
	Few technology-based challenges	“I had to do the first one like 4 times because every time I stopped it didn’t save it.”
Feasibility of intervention	Practical to complete during clinic visits or at home between prenatal visits	“Seems pretty doable to complete 3 (modules) over course of pregnancy.”
Recommendation of intervention	Would recommend to other pregnant people receiving medication for opioid use disorder	“I would recommend it to other women for their sobriety because it helps them.” “Oh yeah, definitely (would recommend), especially if it’s your first time having a baby on Suboxone. This hits the key points, so you at least know the basics of what you want to find out.”

## Data Availability

Not applicable.
